# The Effectiveness and Safety of Mesenchymal Stem Cells in the Treatment of Osteoarthritis: A Systematic Review and Meta-analysis of 28 Randomized Controlled Trials

**DOI:** 10.1155/2022/6151866

**Published:** 2022-10-12

**Authors:** Zhiyong Long, Mingsheng Zhang, Tianqing Zhang, Liuting Zeng, Kailin Yang, Tiejun Yang, Ganpeng Yu, Jun Li, Yang Wu, Hua Chen

**Affiliations:** ^1^Department of Rehabilitation Medicine, Guangzhou Panyu Central Hospital, Guangzhou, China; ^2^Department of Rehabilitation Medicine, South China Hospital of Shenzhen University, Shenzhen, China; ^3^Department of Rehabilitation Medicine, Guangdong Provincial People's Hospital, Guangdong Academy of Medical Sciences, Guangdong Provincial Institute of Geriatrics, Guangzhou, China; ^4^Hunan University of Chinese Medicine, Changsha, Hunan, China

## Abstract

**Objective:**

To evaluate the effectiveness and safety of mesenchymal stem cells (MSCs) in the treatment of osteoarthritis (OA).

**Methods:**

Chinese databases (such as CNKI and SinoMed) and English databases (such as PubMed and Embase) were searched to collect randomized controlled trials (RCTs) of MSCs in the treatment of OA. The retrieval time is from inception to October 10, 2021. The literature was strictly selected according to the inclusion and exclusion criteria, data was extracted, and the quality was evaluated. RevMan 5.3 software was used for meta-analysis. STATA was used to evaluate publication bias. The registration number of this systematic review and meta-analysis is CRD42021277145.

**Results:**

A total of 28 RCTs involving 1494 participants were included. The primary outcomes showed that MSCs may reduce WOMAC pain and VAS at the 3rd-month follow-up [WOMAC pain: -3.81 (-6.95, -0.68), *P* = 0.02. VAS: -1.11 (-1.53, -0.68), *P* < 0.00001], and the effect lasts for at least 12 months [WOMAC pain: -4.29 (-7.12, -1.47), *P* = 0.003. VAS: -1.77 (-2.43, -1.12), *P* < 0.00001]. MSCs may also reduce WOMAC stiffness and physical function at the 6th-month follow-up [WOMAC stiffness: -1.12 (-2.09, -0.14), *P* = 0.03. WOMAC physical function: -4.40 (-6.84, -1.96), *P* = 0.0004], and the effect lasts for at least 12 months [WOMAC stiffness: -0.99 (-1.95, -0.03), *P* = 0.04. WOMAC physical function: -3.26 (-5.91, -0.61), *P* = 0.02]. The improvement of WOMAC pain, VAS, WOMAC stiffness, and WOMAC physical function may be clinically significant. Meanwhile, after the MSC injection, Lequesne had been reduced compared with the control group [-4.49 (-8.21, -0.77), *P* = 0.002]. For adverse events, there is no significant difference in the safety of MSC injection and the control group [1.20 (0.97, 1.48), *P* = 0.09]. The quality of WOMAC physical function and adverse events were moderate.

**Conclusion:**

Based on current evidence, MSCs may be a safety therapy that have a good curative effect in the treatment of OA, the onset time is no later than 3 months, and the time to maintain the curative effect is no less than 12 months. However, these results should be generalized with caution due to the generally low quality of evidence and RCTs.

## 1. Introduction

Osteoarthritis (OA) is a disease involving movable joints characterized by cellular stress and extracellular matrix degradation triggered by microscopic and macroscopic lesions that activate maladaptive repair responses, including proinflammatory pathways of innate immunity [1, 2]. It is estimated that by 2032, 30% of people over the age of 45 will have OA [3, 4]. At present, there is no clinical cure for OA. The main goal of treatment is to control the pain caused by OA, delay the progression of the disease, correct deformity, improve or restore joint function, and strive to improve the quality of life of patients [5, 6]. For example, conventional treatment methods are oral medications to control the condition, local joint injections, physical therapy, or direct joint replacement surgery. Especially for advanced OA, artificial joint prosthesis replacement is the gold standard for the treatment of advanced OA, but the life of the prosthesis is limited, and many complications will occur after the operation [7]. The ideal treatment plan is to improve the patient's clinical symptoms and promote cartilage regeneration [6, 8]. At present, symptomatic treatments such as physiotherapy, auxiliary braces, anti-inflammatory drugs, analgesics, hyaluronic acid (HA), glucocorticoids, arthroscopic debridement, and osteotomy cannot promote cartilage repair and cannot substantially improve OA [6]. Therefore, there is an urgent clinical need for new clinical therapeutic approaches to reduce the development of arthritis and relieve pain.

Mesenchymal stem cells (MSCs) are a type of pluripotent adult stem cells with stem cell characteristics isolated and cultured from the mesoderm and ectoderm of various tissues and organs. They are an important type of stem cell family and the most representative adult stem cells. [9], indicating that MSCs may be of great significance in the treatment of OA and cartilage defects [10]. In recent years, a large number of clinical trials of MSCs in the treatment of OA have been conducted in many countries, accumulating a large amount of conclusive clinical evidence, and a number of meta-analyses and systematic reviews have been published [11–18]. These systematic reviews and meta-analysis showed that compared with the control group, after treatment in the MSCs group, the Western Ontario and McMaster University (WOMAC) score decreased significantly, the visual analogue scale (VAS) decreased significantly, and the knee Lequesne index score decreased significantly. There was no statistically significant difference in the incidence of adverse events between the two groups. However, the above meta-analysis has certain shortcomings. For example, the number of RCTs included is not large, mainly retrospective studies and non-RCT clinical trials. The efficacy and safety of MSCs in the treatment of OA are still unclear. Therefore, in order to explore the therapeutic effect and safety of MSCs in the treatment of OA, this study conducted a systematic review and meta-analysis of the randomized controlled trials (RCT) of MSCs in the treatment of OA, so as to provide evidence support for the application of MSCs in the treatment of OA.

## 2. Materials and Methods

### 2.1. Protocol

This systematic review and meta-analysis were conducted strictly in accordance with the protocol registered in PROSPERO (CRD42021277145) and PRISMA-guidelines (see supplementary materials (available here)) [19].

### 2.2. Literature Search Strategy

The researcher searched VIP Database, SinoMed, Wanfang Database, CNKI, PubMed, Embase, Medline Complete, and Web of Science. The retrieval time is from inception to October 10, 2021. The researchers also searched the Cochrane Library and ClinicalTrials.gov. The search strategy of PubMed and Embase is shown in Table S1 as an example.

### 2.3. Inclusion and Exclusion Criteria

(1) Study design: RCTs without any restrictions on the language, year of publication, and so on. (2) Participants: adult patients diagnosed with OA by recognized standards. (3) Intervention: the experimental group is treated with MSCs, which can be combined with other therapies, and there are no restrictions on cell types and administration methods. The control group used the therapy without MSCs. (4) Outcomes: pain [WOMAC pain (0-20) and VAS (0-10 cm)], stiffness [WOMAC stiffness (0-8)], physical function [WOMAC physical function (0-68)], Lequesne index, and adverse events. The value of minimal clinically important difference (MCID) for VAS for pain was 1.02, WOMAC pain score as 1.79, WOMAC physical function score as 5.13, and WOMAC stiffness score as 0.65 [16]. (5) Exclusion criteria: animal experimental research and basic research, case report, comments, reviews or systematic reviews, and research that has been withdrawn

### 2.4. Literature Screening and Data Extraction

Two reviewers screened the literature separately, extracted the data, and cross-checked [20]. When two researchers have a disagreement, they discuss a joint decision with the third researcher. In the literature screening, first read the title and abstract of the literature, exclude irrelevant literature, and then read the full text of the selected literature to further screen out the final candidate literature. The content of the materials to be finally included in the literature includes (1) title, author, publication time, country, and other information; (2) the characteristics of the research object; and (3) follow-up time, intervention measures, outcome indicators, etc. If some important data are missing from the RCTs, we will try to contact the original authors to obtain the data or to estimate the missing standard deviation according to Cochrane Handbook 6.1.0 [21].

### 2.5. Risk of Bias Assessment

Risk of bias was assessed using the Cochrane Risk of Bias 2.0 tool, which assessed randomization process, deviation from included interventions, missing outcome data, outcome measures, and selective reporting [20]. The evaluation results of each module are obtained according to the module decision road map, and the overall bias evaluation is finally summarized and evaluated, including three levels: “low risk of bias,” “some concerns,” or “high risk of bias.” The evaluation was conducted independently by 2 researchers and then cross-checked. Any differences would be discussed and resolved with the third researcher.

### 2.6. Statistical Analysis

The RevMan 5.3 software provided by the Cochrane Collaboration was used for statistical analysis [22]. Enumeration data were expressed using relative risk (RR) and 95% confidence interval (CI). Measurement data were expressed using the weighted mean difference (WMD) and 95% CI. For meta-analysis, the postintervention data (endpoint data) and change data (difference between endpoint and baseline) of WOMAC and VAS were mixed, and WMD was used for pooled effects according to the Cochrane Handbook 6.1.0 [21]. *Q*-test was used for analysis (test level is *a* = 0.1), combined with *I*^2 to quantitatively evaluate the size of heterogeneity. If *I*^2 < 50% and *P* > 0.1, it can be considered that there is homogeneity among multiple similar studies, and the fixed effects model was used for analysis. If *I*^>50% and *P* < 0.1, the random effects model was used. Subgroup analysis would be carried out based on follow-up time and cell source. STATA was used for publication bias analysis (Harbord method for enumeration data and Egger method for measurement data).

### 2.7. Evidence Quality Assessment

GRADE is currently the most widely used grading system, especially in guidelines. The quality of each outcome measure was assessed by the GRADEprofiler software, which generally began to default to high-quality evidence for RCTs, but there were five factors that downgraded high quality and three factors that upgraded [23]. Downgrading factors included inconsistency, risk of bias (limitation), precision, indirectness, and publication bias. Upgrading factors were mainly large effect sizes, dose-response, and confounding factors. If there is no downgrading factor, it is still high-quality evidence, if there is one downgrading factor, it is moderate-quality evidence, two-level downgrade becomes low-quality evidence, and three-level or more downgrade is very low-quality evidence. Finally, the quality of evidence for each outcome was graded as very low, low, moderate, and high.

## 3. Results

### 3.1. Results of the Search

**Figure 1:**
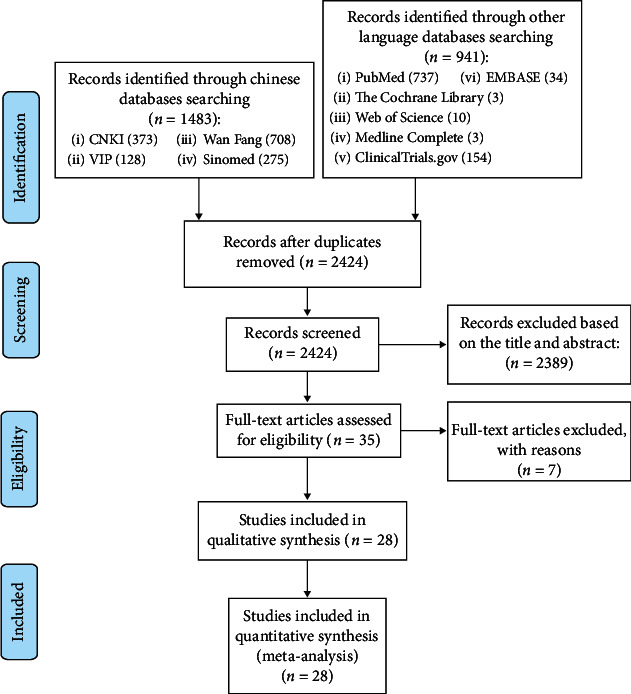
Flow diagram.

A total of 2424 documents were retrieved. After reading the title and abstract, articles that did not fit the topic and duplicates were excluded. Further screening was carried out according to the inclusion and exclusion criteria, and finally 28 RCTs were obtained [24–51], and 7 were excluded [52–58] (Figure 1).

### 3.2. Description of Included Trials

A total of 28 RCTs involving 1494 participants were included. Some RCTs consist of 2 experimental groups, so the control group is divided into 2 equal parts (each containing half the population) to match the two experimental groups, and the matched groups are labeled a and b (as in Kuah et al. [24]). The study characteristics are shown in Table 1.

### 3.3. Risk of Bias Assessment

**Figure 2:**
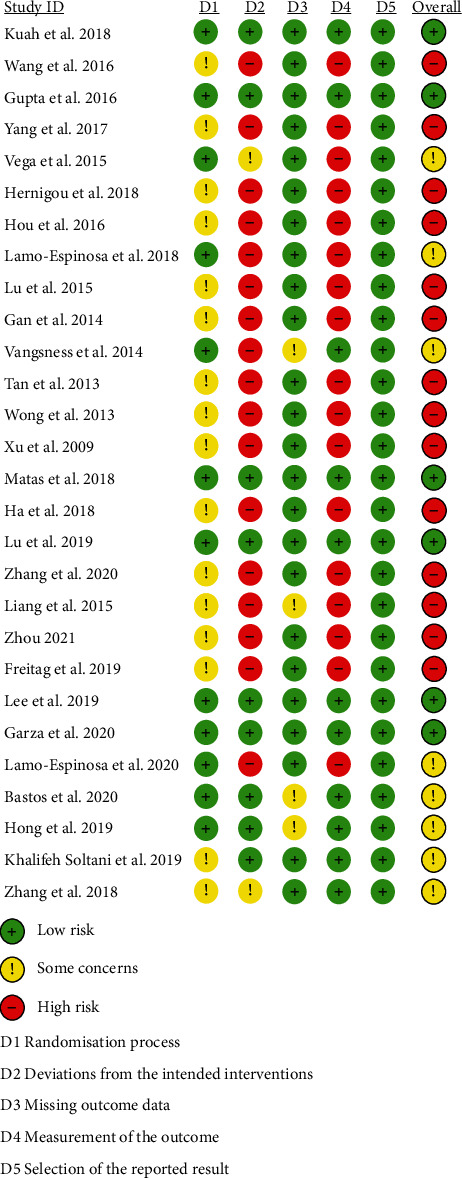
Risk of bias graph.

**Figure 3:**
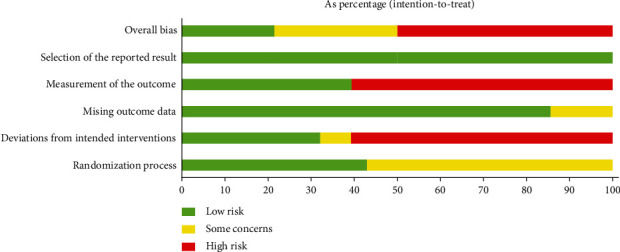
Risk of bias summary.

The risk of bias is assessed and shown in Figures 2 and 3.

### 3.4. Primary Outcomes

#### 3.4.1. Pain

**Figure 4:**
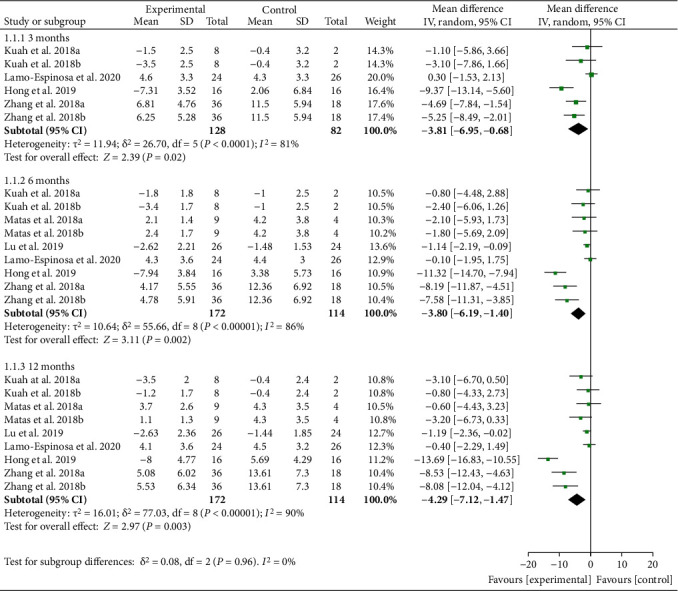
WOMAC pain.

Pain indicators are reflected by VAS and WOMAC pain. Ten RCTs reported WOMAC pain with exact values. The analysis of heterogeneity showed that *I*^2 > 50% and *P* < 0.1 in each subgroup, so the random effects model was adopted. The results showed that after the MSC injection, WOMAC pain had been reduced compared with the control group at the 3rd-month follow-up [-3.81 (-6.95, -0.68), *P* = 0.02], and the effect lasts for at least 12 months [-4.29 (-7.12, -1.47), *P* = 0.003] (Figure 4). The improvement of WOMAC-pain may be clinically significant.

**Figure 5:**
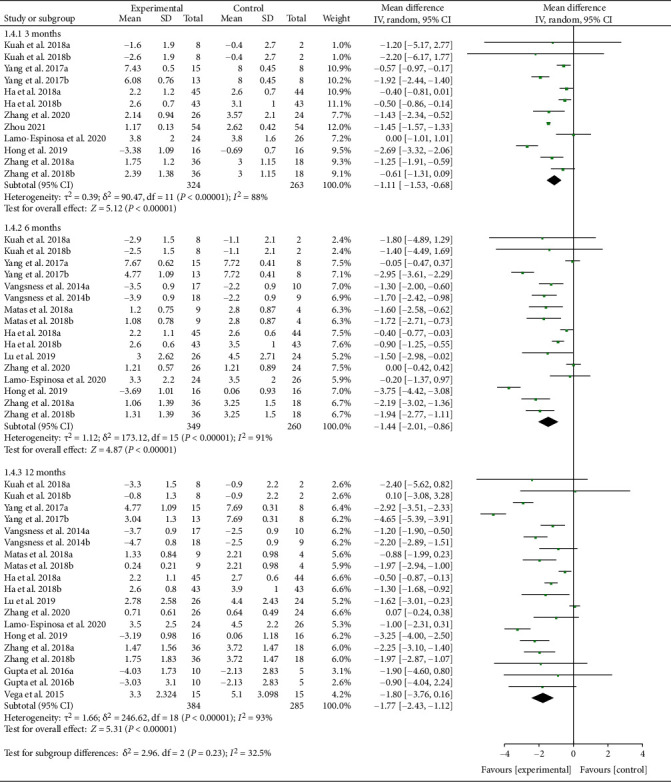
VAS.

Thirteen RCTs reported VAS with exact values. The analysis of heterogeneity showed that *I*^2 > 50% and *P* < 0.1 in each subgroup, so the random effects model was adopted. The results also showed that after the MSC injection, VAS had been reduced compared with the control group at the 3rd-month follow-up [-1.11 (-1.53, -0.68), *P* < 0.00001], and the effect lasts for at least 12 months [-1.77 (-2.43, -1.12), *P* < 0.00001] (Figure 5). The improvement of VAS may be clinically significant.

#### 3.4.2. Stiffness

**Figure 6:**
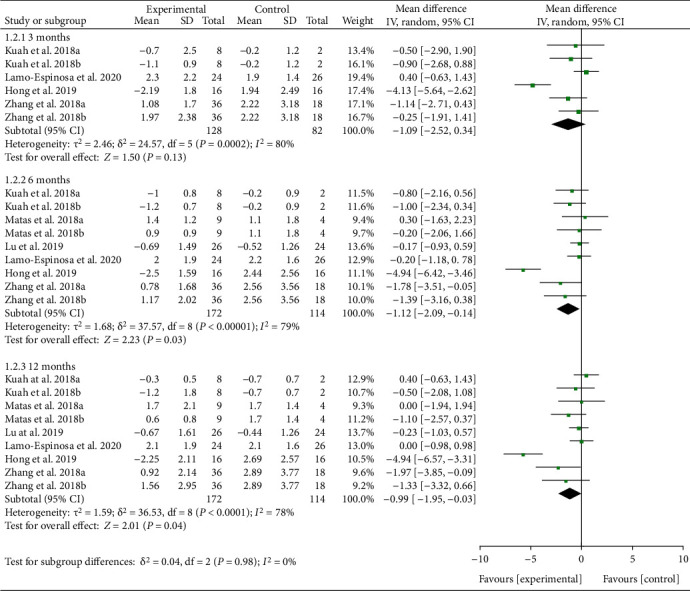
WOMAC stiffness.

Stiffness indicators are reflected by WOMAC stiffness. Seven RCTs reported WOMAC stiffness with exact values. The analysis of heterogeneity showed that *I*^2 > 50% and *P* ≤ 0.1 in each subgroup, so the random effects model was adopted. The results showed that after the MSC injection, WOMAC stiffness had been reduced compared with the control group at the 6th-month follow-up [-1.12 (-2.09, -0.14), *P* = 0.03], and the effect lasts for at least 12 months [-0.99 (-1.95, -0.03), *P* = 0.04] (Figure 6). The improvement of WOMAC stiffness may be clinically significant.

#### 3.4.3. Physical Function

**Figure 7:**
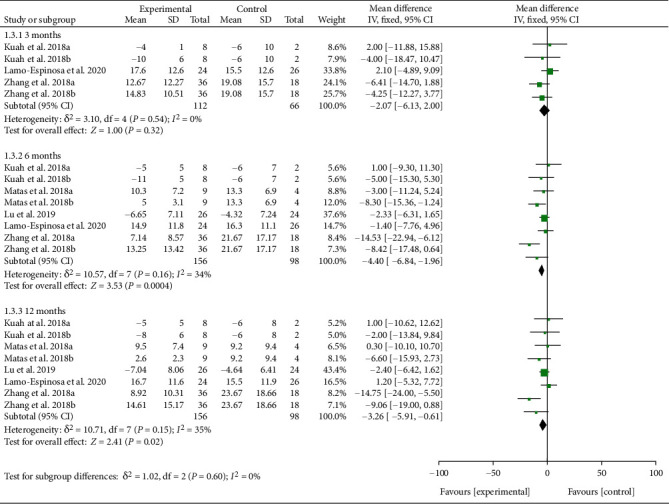
WOMAC physical function.

Physical function indicators are reflected by WOMAC physical function. Six RCTs reported WOMAC physical function with exact values. The analysis of heterogeneity showed that *I*^2 < 50% and *P* > 0.1 in each subgroup, so the fixed effects model was adopted. The results showed that after the MSC injection, WOMAC physical function had been reduced compared with the control group at the 6th-month follow-up [-4.40 (-6.84, -1.96), *P* = 0.0004], and the effect lasts for at least 12 months [-3.26 (-5.91, -0.61), *P* = 0.02] (Figure 7). The improvement of WOMAC physical function may be clinically significant.

#### 3.4.4. Lequesne

**Figure 8:**
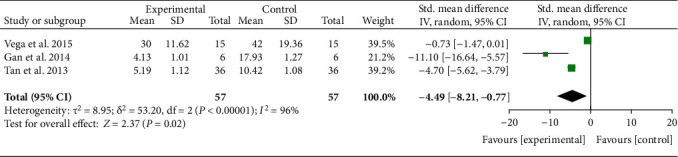
Lequesne.

Three RCTs reported Lequesne with exact values. The analysis of heterogeneity showed that *I*^2 = 96% and *P* < 0.00001, so the random effects model was adopted. The results showed that after the MSC injection, Lequesne had been reduced compared with the control group [-4.49 (-8.21, -0.77), *P* = 0.002] (Figure 8).

### 3.5. Secondary Outcomes

#### 3.5.1. Efficacy of Different Cell Sources on Pain

**Figure 9:**
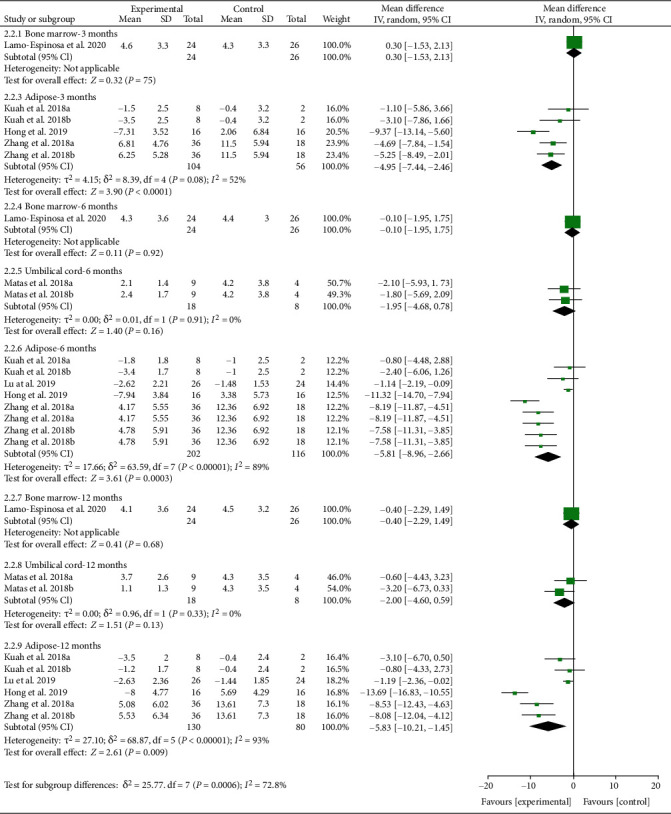
WOMAC pain-different cell sources.

The WOMAC pain and VAS data were divided into subgroups according to follow-up time and cell source. For WOMAC pain, the analysis of heterogeneity showed that *I*^2 > 50% and *P* < 0.1 in almost subgroups, so the random effects model was adopted. (1) For bone marrow derived, only 1 study was involved, and there were no positive findings in this study (*P* > 0.05). (2) For umbilical cord derived, only 1 study was involved, and there were no positive findings in this study (*P* > 0.05). (3) For adipose derived, the results showed that after the MSC injection, WOMAC pain had been reduced compared with the control group at the 3rd-month follow-up [-4.95 (-7.44, -2.46), *P* < 0.0001], and the effect lasts for at least 12 months [-5.83 (-10.21, -1.45), *P* = 0.009] (Figure 9).

**Figure 10:**
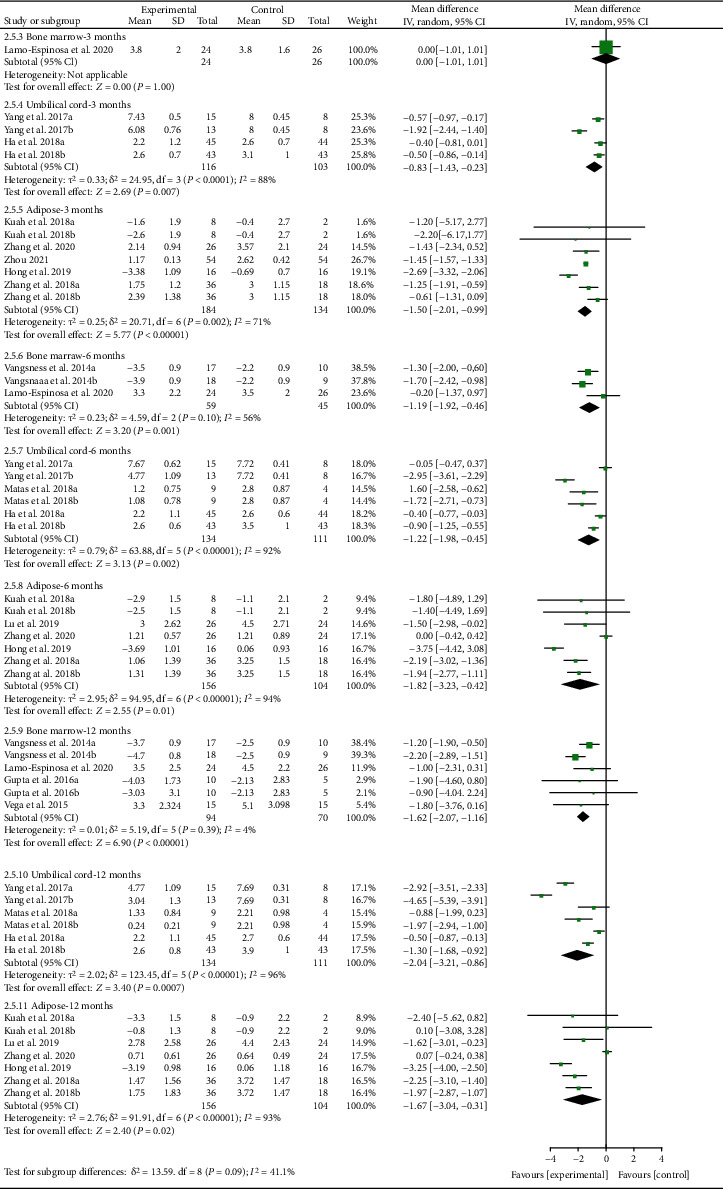
VAS-different cell sources.

For VAS, the analysis of heterogeneity showed that *I*^2 > 50% and *P* < 0.1 in almost subgroups, so the random effects model was adopted. (1) For bone marrow derived, the results showed that after the MSC injection, VAS had been reduced compared with the control group at the 6th-month follow-up [-1.19 (-1.92, -0.46), *P* = 0.001], and the effect lasts for at least 12 months [-1.62 (-2.07, -1.16), *P* < 0.00001]. (2) For umbilical cord derived, the results showed that after the MSC injection, VAS had been reduced compared with the control group at the 3rd-month follow-up [-0.83 (-1.43, -0.23), *P* = 0.007], and the effect lasts for at least 12 months [-2.04 (-3.21, -0.86), *P* = 0.0007]. (3) For adipose derived, the results showed that after the MSC injection, VAS had been reduced compared with the control group at the 3rd-month follow-up [-1.50 (-2.01, -0.99), *P* < 0.00001], and the effect lasts for at least 12 months [-1.67 (-3.04, -0.31), *P* = 0.02] (Figure 10).

#### 3.5.2. Efficacy of Different Cell Sources on Stiffness

**Figure 11:**
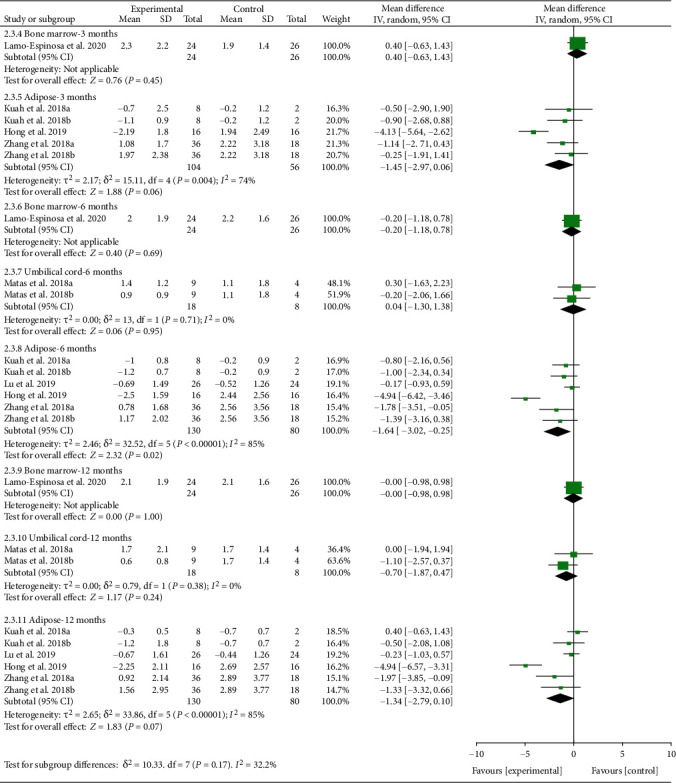
WOMAC stiffness-different cell sources.

The WOMAC stiffness was divided into subgroups according to follow-up time and cell source. The analysis of heterogeneity showed that *I*^2 > 50% and *P* < 0.1 in almost subgroups, so the random effects model was adopted. (1) For bone marrow derived, only 1 study was involved, and there were no positive findings in this study (*P* > 0.05). (2) For umbilical cord derived, only 1 study was involved, and there were no positive findings in this study (*P* > 0.05). (3) For adipose derived, the results showed that after the MSC injection, WOMAC stiffness had been reduced compared with the control group at the 6th-month follow-up [-1.64 (-3.02, -0.25), *P* = 0.02], while the effect was weakened at the 12th-month follow-up [-1.34 (-2.79, 0.10), *P* = 0.07] (Figure 11).

#### 3.5.3. Efficacy of Different Cell Sources on Physical Function

**Figure 12:**
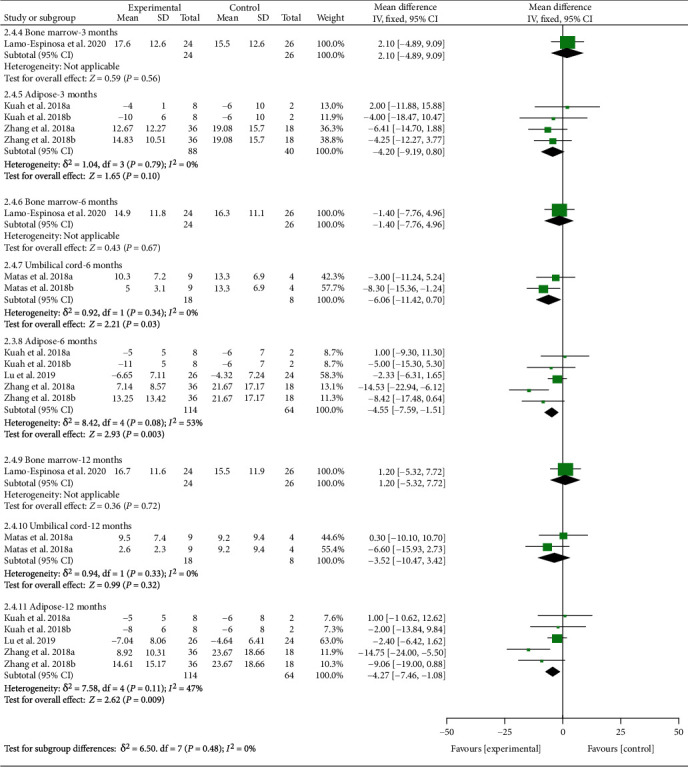
WOMAC physical function-different cell sources.

The WOMAC physical function was divided into subgroups according to follow-up time and cell source. The analysis of heterogeneity showed that *I*^2 < 50% and *P* > 0.1 in almost subgroups, so the fixed effects model was adopted. (1) For bone marrow derived, the results showed that the results of WOMAC physical function did not improve significantly (*P* > 0.05). (2) For umbilical cord derived, the results showed that after the MSC injection, WOMAC physical function had been reduced compared with the control group at the 6th-month follow-up [-6.06 (-11.42, -0.70), *P* = 0.03], while the effect was weakened at the 12th-month follow-up [-3.52 (-10.47, 3.42), *P* = 0.32]. (3) For adipose derived, the results showed that after the MSC injection, WOMAC stiffness had been reduced compared with the control group at the 6th-month follow-up [-4.55 (-7.59, -1.51), *P* = 0.003], and the effect lasts for at least 12 months [-4.27 (-7.46, -1.08), *P* = 0.009] (Figure 12).

### 3.6. Adverse Events

**Figure 13:**
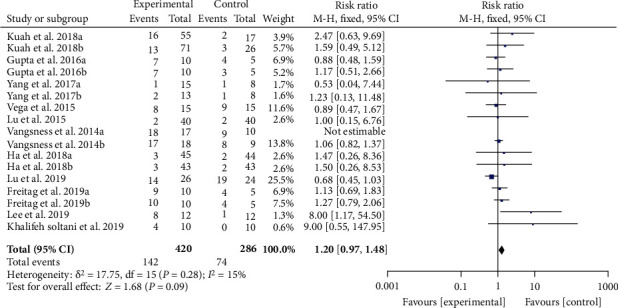
Adverse events.

Ten RCTs reported the number or frequency of adverse events. The heterogeneity test showed that the heterogeneity was low (*I*^2 = 15%, *P* = 0.28); hence, the fixed effects model is used for analysis. The results showed that there is no significant difference in the safety of MSC injection and the control group [1.20 (0.97, 1.48), *P* = 0.09] (Figure 13). The other RCTs such as Hernigou et al. [29], Lamo-Espinosa et al. [31], Matas et al. [38], Garza et al. [46], Lamo-Espinosa et al. [47] all reported no serious adverse events.

### 3.7. Publication Bias of Primary Outcomes

**Figure 14:**
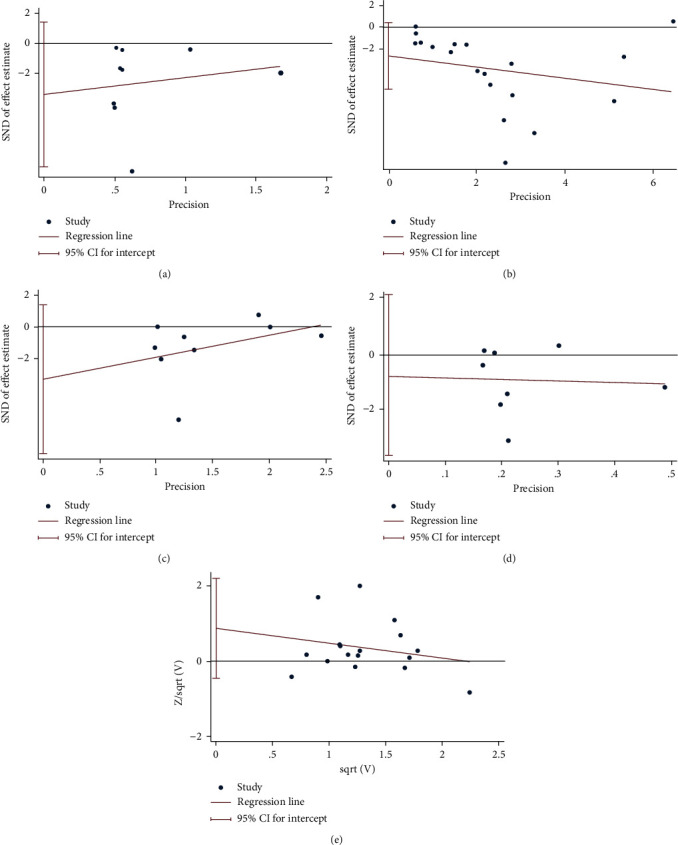
Publication bias: (a) WOMAC pain; (b) VAS; (c) WOMAC stiffness; (d) WOMAC physical function; (e) adverse events.

The primary outcomes were tested for publication bias, and the results showed that these primary outcomes (12 months) are less likely to have publication bias (WOMAC pain: *P* = 0.138; WOMAC stiffness: *P* = 0.142; WOMAC physical function: *P* = 0.536; adverse events: *P* = 0.188), while VAS may have publication bias (*P* = 0.083) (Figure 14).

### 3.8. Quality of Evidence

The evidence at 12-month follow-up was judged to be moderate to very low (Table 2). The quality of WOMAC physical function and adverse events were moderate; the quality of WOMAC pain and WOMAC stiffness were moderate; the quality of VAS was very low (Table 2).

## 4. Discussion

This systematic review and meta-analysis included 28 RCTs involving 1494 participants. In general, intra-articular injection of MSCs may relieve pain (reduce WOMAC pain and VAS) and joint stiffness (reduce WOMAC stiffness) and improve joint function (reduce WOMAC physical function). MSCs may also improve knee arthritis (decrease Lequesne). From the time point of view, the relief of pain by MSCs begins at most the third month after its injection, and the effective time lasts for at least 12 months. The improvement of MSCs on stiffness and physical function starts at most 6 months after injection, and the effective time lasts for at least 12 months. Based on MCID, the changes of WOMAC pain, WOMAC stiffness, WOMAC physical function, and VAS have clinical significance. The WOMAC score scale can effectively reflect the condition of patients before and after treatment, such as the degree of satisfaction of patients, and has high reliability for the assessment of OA. VAS is more sensitive and comparable and can reflect the pain level of patients. The improvement of these results is clinically meaningful, suggesting that MSCs transplantation may be an effective regimen for the treatment of OA. From a cellular point of view, (1) regarding pain, existing studies have shown that bone marrow-derived stem cells begin to take effect at least 12 months after injection, while umbilical cord and adipose derived cells begin to take effect up to 3 months after injection, and the effect lasts at least 12 months. (2) Regarding stiffness, adipose-derived cells begin to take effect up to 6 months after injection, and the effect lasts at least 12 months, while the bone marrow-derived and umbilical cord-derived cells did not show obvious effect. (3) Regarding physical function, umbilical cord and adipose-derived cells begin to take effect up to 6 months after injection, and the effect lasts at least 12 months; while bone marrow-derived cells did not show obvious effect. Safety studies have shown that the adverse events of intra-articular injection of MSCs are similar to those of the control group. It could be considered that the addition of MSCs would not increase the incidence of adverse events.

The quality of evidence assessments shows that the qualities of WOMAC physical function and adverse events were moderate; the qualities of WOMAC pain and WOMAC stiffness were moderate; the quality of VAS was very low. However, because there are few studies related to umbilical cord-derived cells (only 1 RCT reported extractable WOMAC data), more studies on umbilical cord-derived MSCs are needed in the future. In addition, since most of the data reported by RCTs are between 3 months and 12 months, it is impossible to compare the efficacy of 3 months before and after 12 months. Therefore, based on the current evidence, we can only speculate that the onset time of MSCs therapy is no later than 3 months, and the duration of the effect is no earlier than 12 months. More follow-up data are needed in the future to further revise the conclusions.

The dose of RCTs included in this study is basically between 1∗10^7 and 1∗10^8. The clinical data of “Stem Cell Translational Medicine” showed that the Chilean research team used double-dose UC-MSCs to treat knee arthritis more effectively than the single-dose group (cell volume: 2∗10^7). Regardless of single-dose or double-dose treatment, the therapeutic effect of the MSC group was better than that of the hyaluronic acid control group. Only patients treated with MSC had significant improvement in pain and knee joint function (WOMAC-A score). And during the 12th-month follow-up period, no serious adverse events occurred [38]. Another study reported that 12 patients with moderate/severe KOA aged 45-65 received different doses of MSC treatment. The injection doses of the 3 groups were 1∗10^6, 1∗10^7, and 5∗10^7. After 12 months, the pain level and quality of life of all patients have been significantly improved, and at all tested doses, MSC injection is safe, and the test results show that the higher the dose of MSC, the better the effect [59]. In addition, the researchers believe that the number of stem cells used is also important for cartilage regeneration. Jo and other Korean researchers injected adipose MSCs into 18 patients with knee osteoarthritis and divided the patients into a low-dose group (1.0∗10^7), a medium-dose group (5.0∗10^7) and a high-dose group (1.0∗10^8). Studies have shown that the three groups can improve knee joint function and relieve knee pain within 2 years, but only the high-dose group has a statistically significant clinical improvement within 2 years, and the clinical improvement of the middle and low-dose group tends to degenerate after 1 year [60].

Regarding cell sources, the included RCTs mainly involve bone marrow, umbilical cord, adipose, and placenta-derived MSCs. Current research shows that MSCs are easy to accept gene modification, have anti-immune ability, and have strong self-renewal ability [61]. Migliorini et al. found that patients who received bone marrow MSCs (BMSCs) treatment in the early degenerative stage had a good prognosis, significantly improved joint pain and functional scores, and greatly improved the quality of life and recreational activities [62]. A 4-month follow-up study showed that MSCs were effective and safe in the treatment of knee arthritis [63]. In addition, MSCs still have good clinical efficacy and safety in the treatment of patients with mild or moderate knee OA [64]. In the research on the mechanism of BMSCs promoting OA repair, firstly, under certain in vitro induction conditions, BMSCs can differentiate into a variety of cells. Common methods include dexamethasone, sodium *α*-glycerophosphate, and other small molecules to induce MSCs to differentiate into osteoblasts/chondrocytes. Secondly, exosomes (various noncoding RNAs and cytokines, etc.) secreted by BMSCs can promote the repair of osteoarthritis (such as promoting cartilage repair and inhibiting inflammation) [65]. Meanwhile, BMSCs transplantation has many advantages, mainly in the simplicity of acquisition and the value of isolation and culture, easy expansion, and high differentiation potential [66]. However, studies have shown that the differentiation and proliferation ability of BMSCs is unstable during the culture process [67], and the process of extracting bone marrow is traumatic. Some studies compared human cord blood MSCs and BMSCs in vitro induced culture expansion and differentiation potential. They found that both worked well in osteoblast differentiation capacity and could be transplanted as seed cells in the treatment of OA [68, 69]. BMSC has a strong osteogenic potential and has a certain effect on the treatment of OA, but the amount of MSCs in adult bone marrow is small; and due to the limitation of age, the sources of BMSCs are limited, and the clinical effects of BMSCs from different donors are also different [70]. A research found that there is a kind of bone marrow concentrate (BMAC), which shows a good application prospect in the treatment of OA [71]. MSCs in BMAC are rich in a variety of exosomes and paracrine cell growth factors, and in addition to their good repairing effects, they also have immunomodulatory effects, which have potential value for improving the clinical application of OA and regenerative medicine.

Adipose-derived stem cell (ADSC) adipose tissue is also an important source of MSCs. Current studies have shown that ADSCs have the potential to differentiate into mesoderm-derived cells, such as bone/chondrocytes, adipocytes, and muscle cells [72, 73]. A larger RCT involving 110 patients with OA found that microadipose tissue-derived cells and bone marrow concentrate-derived cell injections in patients with knee OA can significantly improve pain and function and thus improve the clinical symptoms of patients. This suggests that cells from these two tissues have a good effect in ameliorating OA, with no significant difference between the two [74]. In addition, ADSCs have many clinical advantages over BMSCs in view of the current preparation and collection of MSCs. For example, ADSC is more convenient and convenient in material acquisition, without ethical restrictions, and has the advantages of strong in vitro expansion ability, low culture difficulty, and strong ability to differentiate into chondrocytes. Therefore, given these advantages, ADSCs are expected to be used in the treatment of OA in the future. Despite the above advantages, the potential clinical problems in the treatment of OA still need to be solved in the future, such as the ability of ADSCs to induce osteogenic differentiation, and its mechanism still need to be further elucidated; in addition, the transplantation of ADSCs into the patient's body requires a material scaffold adapted to human biology as a transplant carrier [75]. Nasb et al. first combined low-intensity pulsed ultrasound and ADSC in the treatment of knee joint OA [76]. The results of this study show that the combination of ADSCs with low-intensity pulsed ultrasound can significantly improve the clinical effect of treatment compared with the comparison of transplantation of ADSCs alone, and the safety is also better than that of transplantation alone. This also provides a reference value for subsequent clinical trials. With the in-depth research on the regulatory mechanism and safety of ADSCs in the future, the treatment of OA with ADSCs will have broader clinical application prospects [76].

A study also reported the therapeutic progress of human cord blood-derived mesenchymal stem cells (hUCB-MSCs) in OA. It is mainly isolated and cultured from umbilical cord blood, and at the same time, it expresses mesenchymal characteristic markers and can be differentiated into bone/cartilage/adipocytes, indicating that Huck-MSCs have multidirectional differentiation potential and high plasticity [77]. Park et al. also used a mixture of cord blood mesenchymal stem cells and hyaluronic acid to repair a woman with knee cartilage injury. One year after the operation, it was found that knee joint function and pain were significantly relieved, and imaging confirmed that there was a normal shape of new cartilage [78]. Song et al. investigated OA patients receiving allogeneic umbilical cord blood mesenchymal stem cell therapy. They were followed up for two years, and the clinical symptoms and quality of life of the patients at the initial 1 and 2 years were significantly improved, and there were no adverse reactions or complications. After at least 2 years of follow-up, hUCB-MSCs implantation is effective in treating knee osteoarthritis [79]. Park et al. followed up to 7 years in patients with OA who were treated with the allogeneic hUCB-MSCs and hyaluronic acid hydrogel complex. In this process, 7 patients underwent treatment were evaluated by arthroscopy and NMR. As a result, none of the 7 patients had adverse reactions, and the symptoms of OA were effectively improved, which proved the effectiveness and safety of hUCB-MSCs [80].

In recent years, clinical research results of using placental MSCs to treat OA have been reported. There is a recent clinical trial for the treatment of knee OA through intra-articular injection of placental MSCs (Trial registration number: IRCT2015101823298N). Twenty patients with symptomatic knee OA were randomly divided into two groups and injected with placental mesenchymal stem cells or saline, respectively. The results showed that the quality of life, daily activities, and exercise of the placental mesenchymal stem cell injection group were significantly improved, and the symptoms of OA were significantly reduced [50].

The advantages of this research are as follows: this systematic review and meta-analysis explores the potential of MSCs as a safe treatment for OA. Compared with previous systematic reviews and meta-analyses [11–18], this meta-analysis conducted a subgroup analysis of RCTs according to source and follow-up time and preliminarily summarized the onset time, duration of efficacy, and tissue origin of MSCs treatment. This meta-analysis also adopted a more stringent risk of bias assessment tool (RoB2) and introduced MCID and found that the efficacy of MSCs may be clinically meaningful.

The limitations of this research were as follows: (1) there are few RCTs involved in some outcomes (such as the WOMAC pain-bone marrow subgroup) so that reliable conclusions cannot be drawn, and more RCTs reporting these outcomes are needed in the future. (2) Due to the source and specific culture conditions of MSCs, the injection site, the injection dose, and the differences between the countries and regions where the research was conducted and the patients included in the research, the RCTs have high heterogeneity and potential risk of bias. This in turn affects the generalization of clinical evidence for the findings. In future clinical trials, more accurate clinical conclusions can be drawn through higher quality study designs. (3) The included RCTs are mainly in Chinese and English, and RCTs in other languages may not be included. Therefore, RCTs reported in other languages may be considered in the future to provide better reference information for clinical treatment. (4) The follow-up time of RCTs is more than 3 months and less than 12 months, resulting in the failure to evaluate the results within 3 months and beyond 12 months. Therefore, RCTs with longer follow-up time and follow-up at more time points are needed in the future.

## 5. Conclusion

At present, OA still seriously threatens human health and quality of life. The development of regenerative medicine and innovative stem cell technology provides a unique opportunity to treat this disease. This systematic review collects RCTs of all types of stem cells to treat OA, in order to make a comprehensive summary, description, and characteristic analysis of these studies. This study shows that MSCs may have a good curative effect in the treatment of OA, the onset time is no later than 3 months, and the time to maintain the curative effect is no less than 12 months. In addition, based on current evidence, it can be considered that ADSC may have a better efficacy because of its early onset of action and a longer duration of efficacy. Regarding safety, MSCs may be considered a safe therapy. However, these results should be generalized with caution due to the generally low quality of evidence and RCTs.

## Figures and Tables

**Table 1:** The characteristics of the included studies.

Study	Trial registration number	Country	Blind	Sample size (female/male)		Intervention		Origin	Dosage	Route of medication	Relevant outcomes	Mean age (years)		Follow-up
				Trial group	Control group	Trial group	Control group					Trial group	Control group	
Kuah et al. [24]	ACTRN12615000439549	Australia	Double-blind	16 (5/11)	4 (3/1)	MSC+placebo	Placebo (cell culture media and cryopreservative)	Human adipose tissue	a: 3.9∗10^6 cells; b: 6.7∗10^6 cells	Intra-articular injection	WOMAC, VAS, adverse events	Low: 50.8 ± 7.29; high: 55.0 ± 5.15	55.0 ± 10.42	12 months
Wang et al. [25]	—	China	Not known	18 (8/10)	18 (7/11)	MSC+sodium hyaluronate	Sodium hyaluronate	Human umbilical cord	2 − 3∗10^7 cells	Intra-articular injection	WOMAC	45-63	42-69	6 months
Gupta et al. [26]	NCT01453738	India	Double-blind	20 (15/5)	10 (10/0)	MSC+placebo	Placebo (Plasmalyte A)	Human bone marrow	a: 2.5∗10^7 cells; b: 5∗10^7 cells	Intra-articular injection	VAS, adverse events	a: 58.10 ± 8.23; b: 57.30 ± 9.45	54.90 ± 8.27	12 months
Yang et al. [27]	—	China	Not known	28 (22/6)	16 (11/5)	MSC+sodium hyaluronate	Sodium hyaluronate	Human umbilical cord	a: 3∗10^7 cells; b: 6∗10^7 cells	Intra-articular injection	VAS, adverse events	a: 70.6 ± 20.1; b: 71.5 ± 16.3	72.2 ± 17.8	12 months
Vega et al. [28]	NCT01586312	Spain	Blind for participants	15 (9/6)	15 (10/5)	MSC+sodium hyaluronate	Sodium hyaluronate	Human bone marrow	4∗10^7 cells	Intra-articular injection	VAS, Lequesne, adverse events	57 ± 9		12 months
Hernigou et al. [29]	—	France	Not known	30 (18/12)	30 (18/12)	MSC+total knee arthroplasty	Total knee arthroplasty	Human bone marrow	2.6∗10^5 cells	Intra-articular injection	Adverse events, other clinical indicators	18-41		8-16 years
Hou et al. [30]	—	China	Not known	92 (52/40)	88 (50/38)	MSC+sodium hyaluronate	Sodium hyaluronate	Human bone marrow	Not known	Intra-articular injection	WOMAC	57 ± 8.3	55 ± 9.2	6 months
Lamo-Espinosa et al. [31, 32]	NCT02123368	Spain	Not known	20 (8/12)	10 (3/7)	MSC+sodium hyaluronate	Sodium hyaluronate	Human bone marrow	a: 1∗10^7 cells b: 1∗10^8 cells	Intra-articular injection	WOMAC, VAS, adverse events	a: 65.9 (59.5, 70.6); b:57.8 (55.0, 60.8)	60.3 (55.1, 61.1)∗	4 years
Lu et al. [33]	—	China	Not known	40 (26/14)	40 (27/13)	MSC+sodium hyaluronate	Sodium hyaluronate	Human bone marrow	Not known	Intra-articular injection	WOMAC, adverse events	55.9 ± 8.1	55.1 ± 6.8	12 months
Gan et al. [34]	—	China	Not known	6 (5/1)	6 (4/2)	MSC+sodium hyaluronate	Sodium hyaluronate	Human umbilical cord	Not known	Intra-articular injection	WOMAC, Lequesne	56.27 ± 7.52	55.96 ± 6.93	12 months
Vangsness et al. [35]	NCT00225095	The US	Double-blind	36 (not kwon)	19 (not kwon)	MSC+sodium hyaluronate	Sodium hyaluronate	Human bone marrow	a: 5∗10^7 cells; b: 1.5∗10^8 cells	Intra-articular injection	VAS, adverse events	—	—	2 years
Tan et al. [34]	—	China	Not known	36 (26/10)	36 (27/9)	MSC+arthroscopy cleanup	Arthroscopy cleanup	Human bone marrow	2 − 3∗10^7 cells	Intra-articular injection	Lequesne	53.37 ± 6.94	53.76 ± 5.68	12 months
Wong et al. [36]	—	The US	Not known	28 (13/15)	28 (14/14)	MSC+sodium hyaluronate	Sodium hyaluronate	Human bone marrow	1.46 ± 0.29∗10^7 cells	Intra-articular injection	Other clinical indicators	36-54	24-54	2 years
Xu et al. [37]	—	China	Not known	20 (not kwon)	20 (not kwon)	MSC+arthroscopic surgery+sodium hyaluronate	Arthroscopic surgery+sodium hyaluronate	Human bone marrow	Not known	Intra-articular injection	WOMAC	44-74		3 years
Matas et al. [38]	NCT02580695	Chile	Double-blind	18 (11/7)	8 (5/3)	MSC+sodium hyaluronate	Sodium hyaluronate	Human umbilical cord	a: 2∗10^7 cells; b: 4∗10^7 cells	Intra-articular injection	WOMAC, VAS, adverse events	a: 56.1 ± 6.8; b: 56.7 ± 4.1	54.8±4.5	12 months
Ha et al. [39]	—	China	Not known	89 (60/29)	86 (63/23)	a: MSC+platelet-rich plasma v.s. platelet-rich plasma; b: MSC v.s. sodium hyaluronate+triamcinolone acetonide		Human umbilical cord	1∗10^7 cells	Intra-articular injection	VAS, adverse events	a: 56.8 ± 6.1 v.s. 55.6 ± 3.6; b: 57.0 ± 3.2 v.s. 56.2 ± 6.7		12 months
Lu et al. [40]	NCT02162693	China	Double-blind	26 (23/3)	26 (23/3)	MSC+sodium hyaluronate	Sodium hyaluronate	Human adipose tissue	5∗10^7 cells	Intra-articular injection	WOMAC, VAS, adverse events	55.03 ± 9.19	59.64 ± 5.97	12 months
Zhang et al. [41]	NCT03955497	China	Not known	14 (9/5)	14 (8/6)	MSC+high tibial osteotomy	High tibial osteotomy	Human adipose tissue	Not known	Intra-articular injection	WOMAC, VAS	61.51 ± 8.80	64.64 ± 9.11	12 months
Liang et al. [42]	—	China	Not known	26 (not known)		MSC	Placebo	Human bone marrow	2∗10^7 cells	Intra-articular injection	WOMAC, adverse events	40-65		4 weeks
Zhou [43]	—	China	Not known	54 (not known)	54 (not known)	MSC+sodium hyaluronate	Conventional therapy	Not known	Not known	Intra-articular injection	WOMAC, VAS	56.05 ± 2.39	55.65 ± 3.56	3 months
Freitag et al. [44]	ACTRN12614000814673	Australia	Not known	20 (9/11)	10 (5/5)	MSC	Conventional therapy	Human adipose tissue	a: 1∗10^8 cells (single injection); b: 1∗10^8 cells (two injection)	Intra-articular injection	WOMAC, adverse events	a: 54.6 ± 6.3; b: 54.7 ± 10.2	51.5 ± 6.1	6 months
Lee et al. [45]	—	South Korea	Double-blind	12 (9/3)	12 (9/3)	MSC	Normal saline	Human adipose tissue	1∗10^8 cells	Intra-articular injection	WOMAC, VAS, adverse events	62.2 ± 6.5	63.2 ± 4.2	6 months
Garza et al. [46]	NCT02726945	The US	Double-blind	26 (11/15)	13 (7/6)	MSC	Placebo	Human stromal vascular fraction	a: 1.5∗10^7 cells; b: 3∗10^7 cells	Intra-articular injection	WOMAC, adverse events	a: 60.5 ± 7.9; b: 59.5 ± 11.7	57.1 ± 9.1	12 months
Lamo-Espinosa et al. [47]	NCT02365142	Spain	Not known	24 (7/17)	26 (10/16)	MSC+platelet-rich plasma	Platelet-rich plasma	Human bone marrow	1∗10^8 cells	Intra-articular injection	WOMAC, VAS, adverse events	40-62	33-70	12 months
Bastos et al. [48]	—	Multi-center	Double-blind	30 (15/15)	17 (8/9)	a: MSC+platelet rich plasma; b: MSC	Corticosteroid	Human bone marrow	Not known	Intra-articular injection	Other clinical indicators	a: 60.8 ± 9.9; b: 55.7 ± 7.8	55.9 ± 13.4	12 months
Hong et al. [49]	ChiCTR1800015125	China	Double-blind, but does not describe the implementation	MSC in left: 8 (7/1); MSC in right 8 (6/2)		MSC+sodium hyaluronate	Sodium hyaluronate	Human adipose tissue	Not known	Intra-articular injection	WOMAC, VAS, adverse events	MSC in left: 53 ± 10.97; MSC in right 51 ± 5.95		12 months
Khalifeh Soltani et al. [50]	IRCT2015101823298N	Iran	Double-blind	10 (not known)	10 (not known)	MSC	Normal saline	Human placenta	0.5 − 0.6∗10^8 cells	Intra-articular injection	VAS, adverse events	35-75		24 weeks
Zhang et al. [51]	—	China	Blind for outcome assessment	72 (56/16)	36 (8/28)	MSC+sodium hyaluronate or MSC only	Sodium hyaluronate	Human adipose tissue	Not known	Intra-articular injection	WOMAC, VAS	a: 57.56 ± 14.06; b: 53.39 ± 12.66	56.89 ± 14.53	3 years

**Table 2:** Quality of evidence.

Outcomes	**Illustrative comparative risks**∗**(95% CI)**		**Relative effect (95% CI)**	**No of participants** **(studies)**	**Quality of the evidence** **(GRADE)**	**Comments**
	Assumed risk	Corresponding risk				
	**Control**	**Adverse event**				
WOMAC pain-12 months		The mean WOMAC pain-12 months in the intervention groups was 4.29 lower (7.12 to 1.47 lower)		286 (9 studies)	⊕⊕⊝⊝ low^1,2^	
VAS-12 months		The mean VAS-12 months in the intervention groups was 1.77 lower (2.43 to 1.12 lower)		669 (19 studies)	⊕⊝⊝⊝ very low^1,2,3^	
WOMAC stiffness-12 months		The mean WOMAC stiffness-12 months in the intervention groups was 0.99 lower (1.95 to 0.03 lower)		286 (9 studies)	⊕⊕⊝⊝ low^1,2^	
WOMAC physical function-12 months		The mean WOMAC physical function-12 months in the intervention groups was 3.26 lower (5.91 to 0.61 lower)		254 (8 studies)	⊕⊕⊕⊝ moderate^1^	
Adverse events	Study population		RR 1.2 (0.97 to 1.48)	706 (17 studies)	⊕⊕⊕⊝ moderate^1^	
	259 per 1000	310 per 1000 (251 to 383)				
	Moderate					
	125 per 1000	150 per 1000 (121 to 185)				

∗The basis for the assumed risk (e.g. the median control group risk across studies) is provided in footnotes. The corresponding risk (and its 95% confidence interval) is based on the assumed risk in the comparison group and the relative effect of the intervention (and its 95% CI). CI: confidence interval; RR: risk ratio; GRADE Working Group grades of evidence. High quality: Further research is very unlikely to change our confidence in the estimate of effect. Moderate quality: Further research is likely to have an important impact on our confidence in the estimate of effect and may change the estimate. Low quality: Further research is very likely to have an important impact on our confidence in the estimate of effect and is likely to change the estimate. Very low quality: We are very uncertain about the estimate. ^1^Downgraded one level due to serious risk of bias (random sequence generation, allocation concealment, blinding, and incomplete outcomes), and most of the data comes from the RCTs with moderate risk of bias. ^2^Downgraded one level due to the probably substantial heterogeneity. ^3^Downgraded one level due to the potential publication bias.

## Data Availability

All data generated or analyzed during this study are included in this published article.

## Glossary

*RCT*: Randomized controlled trial
TCM: Traditional Chinese medicine
RR: Risk ratio
CI: Confidence interval.

## Supplementary Materials

Supplementary Materials PRISMA 2020 checklist: Checklist; Table S1: search Strategies for PubMed and Embase.
