# Supplementary material for: The Effectiveness and Safety of Mesenchymal Stem Cells in the Treatment of Osteoarthritis: A Systematic Review and Meta-analysis of 28 Randomized Controlled Trials
Source: Stem Cells Int. 2022 Oct 12;2022:6151866. doi: 10.1155/2022/6151866 (PMC9581629; doi:10.1155/2022/6151866)
Supplement: Supplementary Materials — PRISMA 2020 checklist: Checklist; Table S1: search Strategies for PubMed and Embase. [file 6151866.f1.pdf]

Table S1. Search Strategies for Pubmed and Embase

**PubMed**	(Mesenchymal stem cells OR Stem Cell, Mesenchymal OR Stem Cells, Mesenchymal OR Mesenchymal Stem Cell OR Bone Marrow Mesenchymal Stem Cells OR Bone Marrow Mesenchymal Stem Cell OR Bone Marrow Stromal Cells OR Bone Marrow Stromal Cell OR Bone Marrow Stromal Cells, Multipotent OR Multipotent Bone Marrow Stromal Cell OR Multipotent Bone Marrow Stromal Cells OR Adipose-Derived Mesenchymal Stem Cells OR Adipose Derived Mesenchymal Stem Cells OR Adipose Tissue-Derived Mesenchymal Stem Cell OR Adipose Tissue Derived Mesenchymal Stem Cell OR Adipose Tissue-Derived Mesenchymal Stem Cells OR Adipose Tissue Derived Mesenchymal Stem Cells OR Mesenchymal Stem Cells, Adipose-Derived OR Mesenchymal Stem Cells, Adipose Derived OR Adipose Tissue-Derived Mesenchymal Stromal Cells OR Adipose Tissue Derived Mesenchymal Stromal Cells OR Adipose-Derived Mesenchymal Stromal Cells OR Adipose Derived Mesenchymal Stromal Cells OR Adipose-Derived Mesenchymal Stem Cell OR Adipose Derived Mesenchymal Stem Cell OR Mesenchymal Stromal Cells OR Stromal Cell, Mesenchymal OR Stromal Cells, Mesenchymal OR Mesenchymal Stromal Cell OR Multipotent Mesenchymal Stromal Cells OR Mesenchymal Stromal Cells, Multipotent OR Multipotent Mesenchymal Stromal Cell OR Mesenchymal Progenitor Cell OR Mesenchymal Progenitor Cells OR Progenitor Cell, Mesenchymal OR Progenitor Cells, Mesenchymal OR Wharton Jelly Cells OR Wharton's Jelly Cells OR Wharton's Jelly Cell OR Whartons Jelly Cells OR Bone Marrow Stromal Stem Cells)<br>AND<br>(Osteoarthritides OR Osteoarthrosis OR Osteoarthroses OR Arthritis, Degenerative OR Arthritides, Degenerative OR Degenerative Arthritides OR Degenerative Arthritis OR Arthrosis OR Arthroses OR Osteoarthrosis Deformans)<br>AND<br>(random* controlled trial [pt] OR controlled clinical trial* [pt] OR randomized [tiab] OR placebo [tiab] OR drug therapy [sh] OR random* [tiab] OR trial* [tiab] OR group* [tiab])<br>NOT<br>(animals [mh] NOT humans [mh])
**EMBASE**	1 Mesenchymal Stem Cells<br>2 'Stem Cell, Mesenchymal'<br>3 'Stem Cells, Mesenchymal'<br>4 'Mesenchymal Stem Cell'<br>5 'Bone Marrow Mesenchymal Stem Cells'<br>6 'Bone Marrow Mesenchymal Stem Cell'<br>7 'Bone Marrow Stromal Cells'<br>8 'Bone Marrow Stromal Cell'<br>9 'Bone Marrow Stromal Cells, Multipotent'<br>10 'Multipotent Bone Marrow Stromal Cell'<br>11 'Multipotent Bone Marrow Stromal Cells'<br>12 'Adipose-Derived Mesenchymal Stem Cells'<br>13 'Adipose Derived Mesenchymal Stem Cells'<br>14 'Adipose Tissue-Derived Mesenchymal Stem Cell'<br>15 'Adipose Tissue Derived Mesenchymal Stem Cell'<br>16 'Adipose Tissue-Derived Mesenchymal Stem Cells'<br>17 'Adipose Tissue Derived Mesenchymal Stem Cells'<br>18 'Mesenchymal Stem Cells, Adipose-Derived'<br>19 'Mesenchymal Stem Cells, Adipose Derived'<br>20 'Adipose Tissue-Derived Mesenchymal Stromal Cells'<br>21 'Adipose Tissue Derived Mesenchymal Stromal Cells'<br>22 'Adipose-Derived Mesenchymal Stromal Cells'<br>23 'Adipose Derived Mesenchymal Stromal Cells'<br>24 'Adipose-Derived Mesenchymal Stem Cell'

	25 'Adipose Derived Mesenchymal Stem Cell'<br>26 'Mesenchymal Stromal Cells'<br>27 'Stromal Cell, Mesenchymal'<br>28 'Stromal Cells, Mesenchymal'<br>29 'Mesenchymal Stromal Cell'<br>30 'Multipotent Mesenchymal Stromal Cells'<br>31 'Mesenchymal Stromal Cells, Multipotent'<br>32 'Multipotent Mesenchymal Stromal Cell'<br>33 'Mesenchymal Progenitor Cell'<br>34 'Mesenchymal Progenitor Cells'<br>35 'Progenitor Cell, Mesenchymal'<br>36 'Progenitor Cells, Mesenchymal'<br>37 'Wharton Jelly Cells'<br>38 'Whartons Jelly Cells'<br>39 'Bone Marrow Stromal Stem Cells'<br>40 Whartons Jelly Cells<br>41 Bone Marrow Stromal Stem Cells<br>42 or/1-41<br>43 'Osteoarthritis'/exp<br>44 'Osteoarthritides' or 'Osteoarthrosis' or 'Osteoarthroses'<br>45 'Degenerative Arthritides' OR 'Degenerative Arthritis' OR 'Osteoarthrosis Deformans'<br>46 or/43-45<br>47 'randomized controlled trial'<br>48 'single blind procedure' or 'double blind procedure'<br>49 'crossover procedure'<br>50 or/47-49<br>51 42 and 46<br>52 50 and 51
